# Factors Affecting Life-Space Mobility of Home-Care Older Adults Receiving Home-Visit Rehabilitation Using Path Analysis: A Cross-Sectional Multicenter Study

**DOI:** 10.7759/cureus.81486

**Published:** 2025-03-31

**Authors:** Yuta Sugita, Takeshi Ohnuma, Eisuke Kogure, Tsuyoshi Hara

**Affiliations:** 1 Department of Rehabilitation, Nishinasuno Marronnier Visiting Nurse Station, Nishinasuno General Home Care Center, Nasushiobara, JPN; 2 Home-Visit Nursing Division, Rehabilitation Progress Center Incorporated, Tokyo, JPN; 3 Department of Rehabilitation, Acinara Home Visit Nurse Station, GOJO Incorporated, Tokyo, JPN; 4 Department of Physical Therapy, School of Health Sciences, International University of Health and Welfare, Otawara, JPN

**Keywords:** environment, home-care services, home-visit rehabilitation, mobility limitation, rehabilitation

## Abstract

Aim: Life-space mobility (LSM) limitations are a significant concern associated with facility admission, mortality, and quality of life in older adults. Home-visit rehabilitation (HR) users are particularly vulnerable to LSM restrictions, making its maintenance and improvement a priority in this population. This study aimed to assess LSM using the life-space assessment (LSA) and expand existing conceptual models for independent community-dwelling older adults in Japan. Additionally, we analyzed factors influencing LSM in HR users.

Methods: This multicenter cross-sectional study included 105 HR users, comprising 56 men (53.3%) and 49 women (46.7%); mean age 78.5 ± 7.7 years, from urban and rural areas between August 2020 and October 2022. Motor function (grip strength, 30-second chair stand test, CS-30), psychological factors (Self-Efficacy Scale on Going out among community-dwelling Elderly, SEGE), activities of daily living (ADL) ability (functional independence measure, FIM), instrumental ADL (IADL) ability (Frenchay Activities Index, FAI), and environmental factors (home and communication environment, living alone, and day service use frequency), which have been reported in previous studies, were collected for parameters related to LSA. Path analysis examined associations between these factors and LSA.

Results: LSA revealed direct effects on FAI (β = 0.344), FIM-motor score (β = 0.261), living alone (β = -0.196), and day service use frequency (β = 0.184). Indirect effects were observed in CS-30 (β = 0.220), SEGE (β = 0.085), and sex (β = -0.087). The model demonstrated good fit (goodness-of-fit index, GFI, 0.956; adjusted GFI, 0.910; comparative fit index, 1.000; root mean square error of approximation, 0.000).

Conclusion: ADL, IADL, and environmental factors directly affect LSA in home-care older adults using HR, while motor function, psychological factors, and sex have indirect effects. These findings highlight the importance of considering these relationships when designing rehabilitation strategies to support LSM. Future research should examine broader populations, additional variables, and longitudinal data to refine interventions for HR users.

## Introduction

Life-space mobility (LSM) is defined as the extent of movement within a given time period [1]. Limitations in LSM are associated not only with activities of daily living (ADL) [2,3] and instrumental ADL (IADL) [2,3] disability but also with hospitalization [4], mortality [5], and quality of life decline [6]. Therefore, assessing and improving LSM are crucial for older adults to maintain their independence and live in their familiar environment at home.

The life-space assessment (LSA) is a widely used tool for evaluating LSM [3]. It is considered a valuable indicator in independent community-dwelling and home-care older adults [7]. Factors associated with LSA in community-dwelling older adults include demographic characteristics, such as age [8,9] and sex [8,10]; physical function, such as grip strength, GS [11,12], lower limb function [8], lower limb muscle strength [9], walking status [13], and time up and go test (TUG) [14]; ADL ability [9,14,15]; IADL ability [14,16-18], such as community living and recreational/leisure activities [19] and social participation status [20,21]; and environmental factors, such as number of barriers [22], driving [23], having a roommate [14], and having a car driver [20,24]. Considering these diverse influences, a comprehensive approach integrating multiple factors is necessary to assess LSA effectively.

In Japan, studies have explored structural relationships among parameters affecting LSA [25,26]. One study [25] found that health status, physical function, and environmental factors directly influence LSA in independent community-dwelling older adults, while hobby activities exert an indirect effect through physical function. Another study [26] reported that demographic characteristics, psychological factors, and TUG directly influence LSA, whereas lower limb function has an indirect effect. These studies [25,26] provide a conceptual framework for understanding the pathways through which various factors interact to influence LSA, highlighting the importance of identifying both direct and indirect determinants.

However, to our knowledge, no studies have developed a model to clarify the relationships between LSA parameters in home-visit rehabilitation (HR) users. Previous research [27,28] suggests that HR users exhibit lower LSA and restricted LSM, making them more likely to face challenges in continuing to live at home. It is, therefore, critical to focus on this population.

Previous studies [25,26] have examined independent community-dwelling older adults without assessing ADL or IADL abilities. Given the range of relevant factors [8-24] influencing LSA, a more comprehensive analysis is needed, incorporating ADL abilities, IADL abilities, and environmental factors. Additionally, as these studies were conducted at a single center [25,26], their generalizability is limited. Multicenter studies including participants from diverse geographical areas and residential settings are needed for broader findings.

This study aimed to develop a model for LSA in HR users and examine how parameters previously associated with LSA in independent community-dwelling and home-care older adults affect this group. Therefore, we used an LSA model of independent community-dwelling adults reported in Japan as a reference [25,26]. Since the previous LSA models [25,26] did not consider ADL, IADL, and environmental factors, we created a more comprehensive model of LSA for HR users by adding these factors and examined the differences between this model and the previous LSA models. By clarifying these relationships and their effects, this study aims to provide insights into improving LSA among HR users. The results of this study may help plan programs to improve LSA in HR and identify areas of focus.

## Materials and methods

Study design

This multicenter cross-sectional study examined HR users at two home nursing stations in urban and rural Japan. Previous studies [25,26] investigated LSA models based on health status, physical function, environmental factors, hobbies, and demographic information. In addition to these factors [25,26], this study collected data on ADL ability, IADL ability, and psychological factors to explore their association with LSA in HR users. A previous study [25] assessed health status based on the presence of greater than or equal to three medications, regular hospital visits, and hospitalization in the last year. The participants in this study already had medical conditions requiring regular hospital visits. Older adults receiving home care often have multiple comorbidities and use several medications [29]. Therefore, the variables used in the previous study were expected [25] to show less variability among this study’s participants. Accordingly, this study prioritized factors that HR could support, excluding health status, to validate the LSA model. The study parameters were measured by physiotherapists or occupational therapists in charge of the participants, who were trained in the measurement methods before the study.

Participants

HR users registered at the two home nursing stations between August 2020 and October 2022 were eligible. All HR users who did not meet the exclusion criteria were invited to participate. Exclusion criteria included hospitalization, age <65 years, discontinued HR use, nearing the end of HR, or residence in a nursing home. Also excluded were those unable to provide consent, those deemed ineligible by the therapist, and those with missing values. Those deemed ineligible by the therapist were those unable to follow verbal instructions or communicate due to conditions such as dementia, ventilator dependency, or stroke, those with deteriorating conditions, and those unable to participate due to difficulty in establishing rapport and mental instability.

The study was approved by the International University of Health and Welfare Graduate Ethics Review Committee (approval number 19-Io-237). All participants or their primary caregivers were informed of the study’s purpose and content, and written informed consent was obtained. The study adhered to the principles of the Declaration of Helsinki.

All participants received physician-directed HR instructions, including HR times and programs, exercise therapy, and home living support. They were also instructed on ADL and IADL exercises, environmental adjustments (e.g., selecting home support equipment), and caregiving for housemates.

Evaluation of LSA

The primary outcome was LSA [3], assessed based on living space, mobility frequency, and independence over the previous month. The LSA score was calculated by multiplying the five score levels for each life space (1 = home, 2 = out of home, 3 = neighborhood, 4 = town, 5 = out of town) by mobility frequency (1 = less than one day per week, 2 = one to three days per week, 3 = four to six days per week, 4 = daily) and independence (1 = needs help from others, 1.5 = uses assistive devices, 2 = independent). Scores ranged from 0 to 120.

Evaluation of other measurements

Relevant parameters for physical function and environmental factors were collected based on the LSA model [25,26] for independent community-dwelling older adults. Additionally, demographic information, including age, sex, primary illness, HR duration (months), HR intervention frequency (times/week), and number of HR staff, was extracted from medical records.

Beyond the existing conceptual model, additional parameters related to motor function, psychological factors, ADL ability, IADL ability, and environmental factors influencing LSA were collected with reference to previous studies.

Motor function was assessed using GS and the 30-second chair stand test (CS-30). The conceptual LSA model for healthy community-dwelling older adults includes assessments of GS, the TUG test, and the one-leg stand test [25]. While GS can be easily measured in home settings, many participants requiring ADL assistance might be unable to perform TUG or one-leg stand tests, particularly due to high variability in the latter [27]. Therefore, CS-30, which can be evaluated as lower limb function, was used instead. GS was measured twice in a seated resting position using a digital GS meter (GRIP-D-TKK5401; Takei, Niigata, Japan), with the maximum value recorded. CS-30 [30] measured the number of chair stands performed in 30 seconds, recorded twice, with the highest value used.

Psychological factors, including self-efficacy, were assessed using the Self-Efficacy Scale on Going out among community-dwelling Elderly (SEGE) [31]. This scale comprises six items (e.g., I can go out without any particular reason), rated on a 4-point scale (1 = not confident to 4 = confident), with scores ranging from 6 to 24.

ADL ability was assessed using the functional independence measure (FIM) [32], which includes FIM motor (FIM-M) and FIM cognitive (FIM-C) items. Each item is rated on a 7-point scale based on ADL independence. Total scores, FIM-M, and FIM-C were used, with total scores ranging from 18 to 126.

IADL ability was assessed using the Japanese version of the Frenchay Activity Index (FAI) [33], which consists of 15 questions evaluating activity frequency over the past three months on a 4-point scale. Scores ranged from 0 to 45.

Environmental factors were assessed using the Home and Community Environment (HACE) scale [34], which comprises 35 items across six domains. In the domains of home mobility, community mobility, and attitudes, higher scores indicate greater barriers. In the domains of transportation factors, basic mobility devices, and communication devices, higher scores reflect greater facilitating factors. A total score was calculated across all six domains. Additionally, human support and social resource use were assessed by determining whether participants lived alone and recording the frequency of day service use (number of times/week).

Statistical analyses

Collected parameters included age and sex (male: 1, female: 0). Categorical variables were compared using the chi-square test, while quantitative variables were analyzed using the Mann-Whitney U test. Spearman's rank correlation coefficient was used to assess correlations between LSA and collected parameters, as well as multicollinearity among parameters.

**Figure 1:**
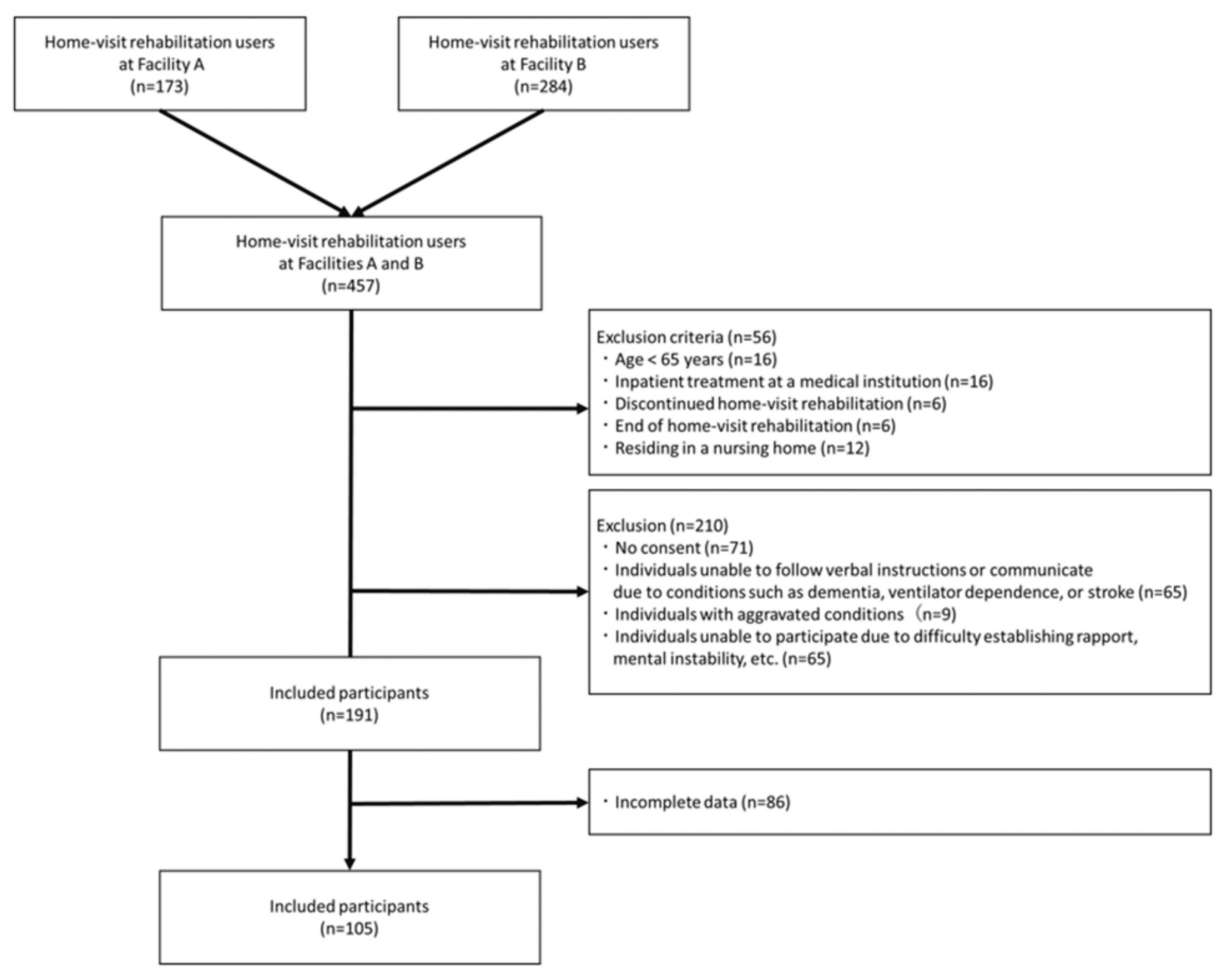
Participant selection flowchart

A hypothetical model (Figure 1) was then developed to examine the relationships between factors influencing each life space, and a path analysis was conducted to determine interrelationships among these factors. Model fit was evaluated using the goodness-of-fit index (GFI), adjusted GFI (AGFI), comparative fit index (CFI), and root mean square error of approximation (RMSEA). Based on previous studies [35], values of GFI >0.90, AGFI >0.90, CFI >0.90, and RMSEA <0.05 indicated good model fit.

The direct, indirect, and total effects of each factor on LSA were further analyzed. Paths and parameters with low standardized path coefficients that lacked statistically significant associations were excluded to derive the final model. Statistical analyses were performed using IBM Statistical Package for the Social Sciences (SPSS) Statistics, version 29.0 (IBM Corp., Armonk, NY) and IBM SPSS Amos, version 29.0 Graphics (IBM Corp., Armonk, NY). Statistical significance was set at 5%.

## Results

Participant selection

Of the 457 home-care patients receiving HR at urban and rural home nursing stations, 56 met the exclusion criteria, and 71 did not consent. And of those deemed ineligible by the therapists, 65 were unable to participate due to conditions such as dementia, ventilator dependence, or stroke that prevented them from following verbal instructions or communicating, nine had deteriorating conditions, and 65 had difficulty establishing rapport and faced mental instability, resulting in 191 participants. After excluding another 86 participants with missing values, 105 were included in the final analysis (Figure 1, Table 1).

**Table 1:** Participant characteristics Measurement variables are expressed as the mean ± standard deviation and the actual value (percentage) HR: home-visit rehabilitation

Home-visit rehabilitation users	All participants (n = 105)	
		Age (years)	78.5 ± 7.7	
65-74 years	69 (65.7)	
≥75 years	36 (34.3)	
Sex		
Male	56 (53.3)	
Female	49 (46.7)	
Facility		
A (urban)	67 (63.8)	
B (rural)	38 (36.2)	
Primary disease		
Cerebrovascular disease	26 (24.8)	
Musculoskeletal disease	23 (21.9)	
Neuromuscular disease	31 (29.5)	
Cardiovascular disease	4 (3.8)	
Respiratory disease	5 (4.8)	
Cancer	6 (5.7)	
Cognitive/psychiatric disease	1 (1.0)	
Others	9 (8.6)	
HR information		
HR duration (months)	33.6 ± 37.6	
HR intervention frequency (times/week)	1.4 ± 0.6	
Number of HR staff	1.4 ± 0.6	

HR user demographics and characteristics (105 participants)

Table 2 presents univariate analyses of collected parameters by sex and age. GS was significantly higher in men and in the 65-74 age group, while FAI was significantly higher in women. No other parameters showed significant differences.

**Table 2:** Comparative analysis results This table shows the results of a comparison test by age and sex (male: 1, female: 0) Significance level: ^*^p < 0.05; ^**^p < 0.001 ^a^Measurement variables are expressed as medians (interquartile range) and compared using the Mann-Whitney U test ^b^Categorical variables are expressed as the actual values (percentages) and compared using the chi-square test CS-30: 30-second chair stand test; SEGE: Self-Efficacy scale on Going out among community-dwelling Elderly; ADL: activities of daily living; FIM: functional independence measure; IADL: instrumental activities of daily living; FAI: Frenchay Activity Index; HACE: home and community environment

Home-visit rehabilitation users	All participants (n = 105)	65-74 years (n = 69)	≥75 years (n = 36)	U-value	Chi-square value	p value	Female (n = 49)	Male (n = 56)	U-value	Chi-square value	p value	
												Primary outcome												
Life-space assessment scores^a^ (points)	29 (3-100)	29 (3-100)	29 (6-51)	1,227	-	0.919	30 (6-73.5)	29 (3-100)	1,157	-	0.167	
Physical performance												
Grip strength^a^ (kg)	18.5 (7.5-43.9)	17.3 (7.5-43.8)	21.9 (9-43.9)	1,584.5	-	0.021^*^	15.5 (7.5-27)	23.3 (8.5-43.9)	2,157	-	<0.001^**^	
CS-30^a ^(times)	0 (0-21)	3 (0-21)	0 (0-16)	1,123.5	-	0.385	4 (0-16)	0 (0-21)	1,248	-	0.387	
SEGE^a^ (points)	8 (6-23)	7 (6-23)	8 (6-18)	1,330	-	0.530	8 (6-21)	7.5 (6-23)	1,366	-	0.968	
ADL ability												
FIM-motor^a^ (points)	76 (28-90)	76 (32-90)	76 (28-90)	1,230.5	-	0.938	77 (32-90)	75.5 (28-90)	1,205.5	-	0.285	
FIM-cognitive^a ^(points)	34 (12-35)	34 (21-35)	35 (12-35)	1,428	-	0.186	34 (23-35)	34 (12-35)	1,264.5	-	0.467	
FIM-total score^a^ (points)	110 (40-125)	110 (65-124)	111 (40-125)	1,309	-	0.651	110 (65-124)	108 (40-125)	1,200	-	0.269	
IADL ability												
FAI score^a^ (points)	6 (0-35)	6 (0-35)	7.5 (0-30)	1,348	-	0.472	10 (0-35)	4 (0-25)	903	-	0.002^*^	
Environment factor												
Living alone^b ^(yes)	23 (21.9)	16 (23.2)	7 (19.4)	-	0.194	0.660	13 (26.5)	10 (17.9)	-	1.149	0.284	
HACE facilitator^a^ (points)	7 (0-15)	8 (1-15)	7 (1-15)	1,280	-	0.796	8 (1-15)	7 (1-14)	1,335	-	0.811	
HACE barrier^a^ (points)	4 (0-14)	4 (0-14)	4 (0-8)	1,204	-	0.796	3 (0-9)	5 (0-14)	1,647	-	0.075	

Spearman's rank correlation coefficient results

Table 3 displays correlations between LSA and collected parameters. LSA was significantly correlated with CS-30 (rs = 0.353, p < 0.001), SEGE (rs = 0.443, p < 0.001), FIM-M (rs = 0.440, p < 0.001), FIM-C (rs = 0.250, p < 0.001), FIM-total score (rs = 0.441, p < 0.001), FAI (rs = 0.508, p < 0.001), and day-care service use frequency (rs = 0.192, p < 0.05). No parameter had a correlation coefficient ≥0.8.

**Table 3:** Results of the correlation analyses between life-space assessment scores and other measurements Significance level: ^*^p < 0.05; ^**^p < 0.001 Spearman’s rank correlation coefficient was used for the correlation analysis CS-30: 30-second chair stand test; SEGE: Self-Efficacy scale on Going out among community-dwelling Elderly; FIM: functional independence measure; FAI: Frenchay Activity Index; HACE: home and community environment

Home-visit rehabilitation users: all participants (n = 105)	Spearman’s rank correlation coefficient	p value
Age	-0.065	0.513
Sex	-0.135	0.168
Grip strength (kg)	0.089	0.366
CS-30 (times)	0.353	<0.001^**^
SEGE (points)	0.443	<0.001^**^
FIM-motor (points)	0.440	<0.001^**^
FIM-cognitive (points)	0.250	<0.001^**^
FIM-total score (points)	0.441	<0.001^**^
FAI score (points)	0.508	<0.001^**^
HACE facilitators (points)	-0.112	0.257
HACE barriers (points)	0.148	0.133
Home mobility scores (points)	0.065	0.508
Community mobility scores (points)	0.154	0.117
Transportation factors scores (points)	0.145	0.140
Attitude scores (points)	-0.069	0.487
Basic mobility devices scores (points)	-0.145	0.139
Communication devices scores (points)	0.057	0.560
Living alone	-0.011	0.091
Day-care service use frequency (times/week)	0.192	0.050^*^

Structural equation modeling results

Examination of Hypothetical Models

Using a model of LSA in independent community-dwelling older people as reference [25,26], motor function (GS), environmental factors (living alone, HACE facilitator/barrier factors, and frequency of day service use), and psychological factors (SEGE) were assumed to influence LSA directly. In contrast, lower limb function (CS-30) was assumed to have an indirect effect. Additionally, ADL ability [9,14,15] (FIM-M and FIM-C) and IADL ability [14,19-21] (FAI), previously associated with LSA, were included as direct factors. The psychological factor (SEGE) self-efficacy is also an important factor [36] related to behavioral choices [37] and activities [9] and has been reported as one of the determinants in LSA. In particular, studies with wheelchair users have reported that self-efficacy is related to LSA via movement ability [38]. Based on these findings, psychological factors (SEGE) were hypothesized to influence LSA indirectly via ADL ability (FIM-M) and IADL ability (FAI).

**Figure 2:**
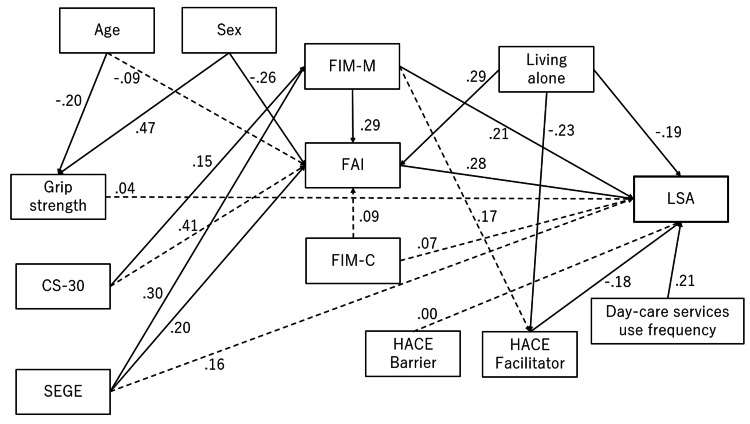
Hypothetical model of the factors that affect life-space assessment in users of home-visit rehabilitation Solid lines indicate a p value of <0.05. The dotted lines indicate that the path coefficients were not significant Model fit: goodness-of-fit index, 0.860; adjusted goodness-of-fit index, 0.772; comparative fit index, 0.752; root mean square error of approximation, 0.097 FIM-M: functional independence measure motor; FAI: Frenchay Activity Index; LSA: life-space assessment; CS-30: 30-second chair stand test; FIM-C: functional independence measure cognitive; SEGE: Self-Efficacy scale on Going out among community-dwelling Elderly; HACE: home and community environment

Based on the above hypotheses, a model was developed and examined. The hypothetical model demonstrated poor fit (GFI = 0.860, AGFI = 0.772, CFI = 0.752, RMSEA = 0.097) (Figure 2).

Consideration in the Final Model

**Figure 3:**
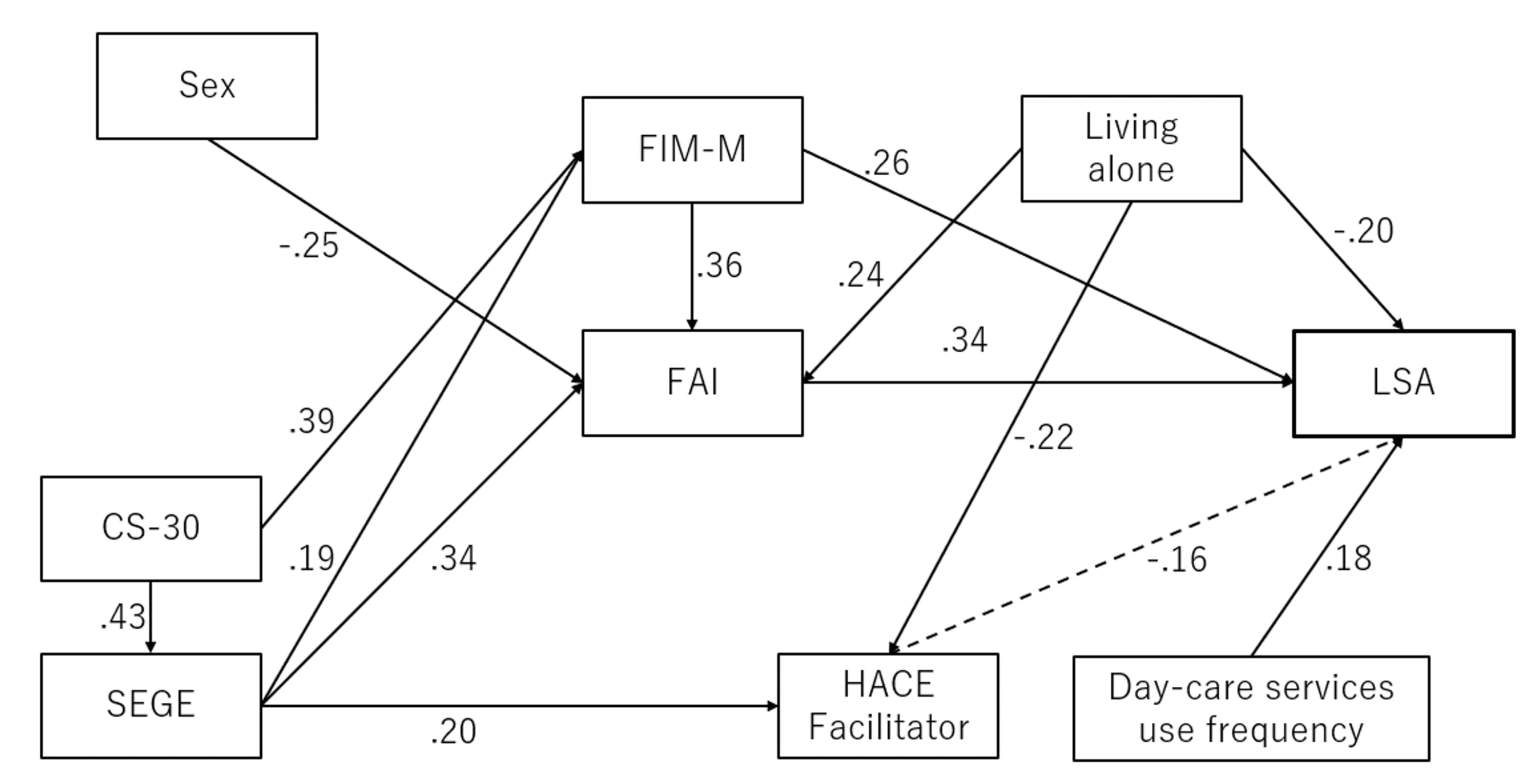
Final model of the factors that affect life-space assessment in users of home-visit rehabilitation Solid lines indicate a p value of <0.05. The dotted lines indicate that the path coefficients were not significant Model fit: goodness-of-fit index, 0.956; adjusted goodness-of-fit index, 0.910; comparative fit index, 1.000; root mean square error of approximation, 0.000 FIM-M: functional independence measure motor; FAI: Frenchay Activity Index; LSA: life-space assessment; CS-30: 30-second chair stand test; SEGE: Self-Efficacy scale on Going out among community-dwelling Elderly; HACE: home and community environment

The model was refined by removing nonsignificant paths. In the final model, LSA was directly affected by FAI (standardized β = 0.344), FIM-M (standardized β = 0.261), living alone (standardized β = -0.196), and day service use frequency (standardized β = 0.184). The CS-30 (standardized β = -0.196) was directly affected. CS-30 (standardized β = 0.220), SEGE (standardized β = 0.160), and sex (standardized β = -0.087) indirectly affected LSA. The final model demonstrated excellent fit (GFI = 0.956, AGFI = 0.910, CFI = 1.000, RMSEA = 0.000) (Figure 3, Table 4).

**Table 4:** Correlation of each variable with life-space assessment scores in the final model. The results of the path analysis in the final model are displayed CS-30: 30-second chair stand test; SEGE: Self-Efficacy scale on Going out among community-dwelling Elderly; FIM: functional independence measure; FAI: Frenchay Activity Index; HACE: home and community environment

Home-visit rehabilitation users: all participants (n = 105)	Standardized direct effect	Standardized indirect effect	Standardized total effect
CS-30 (times)	-	0.220	0.220
SEGE (points)	-	0.160	0.160
FIM-motor (points)	0.261	0.124	0.385
FAI score (points)	0.344	-	0.344
HACE facilitators (points)	-0.160	-	-0.160
Living alone (yes)	-0.196	0.118	-0.077
Sex (male: 1)	-	-0.087	-0.087
Day-care service use frequency (times/week)	0.184	-	0.184

## Discussion

This study found that LSA in HR users was directly influenced by FAI, FIM-M, living alone, and frequency of day service use. Additionally, CS-30, sex, and SEGE indirectly influenced LSA through IADL, ADL, and environmental factors. These findings suggest that interventions focusing on ADL, IADL, and environmental adjustments may enhance LSA in HR users.

To our knowledge, this is the first comprehensive analysis of factors affecting LSA in HR users, incorporating urban and rural settings and multiple parameters identified in previous studies. A prior study using structural equation modeling [25] reported that environmental factors directly influence LSA, aligning with our findings. A well-developed residential environment [39,40] and convenient transportation [41,42] have been linked to increased physical activity in independently living older adults. Similarly, household composition [20,43] and day service use [44] have been associated with LSA in homebound older adults. Our results reinforce these associations and highlight the direct effects of living alone and day service use on LSA.

However, the expected influence of HACE facilitator factors on LSA was not observed. Previous findings [28] may explain this, indicating lower LSA among urban HR users than their rural counterparts. Urban HR users may function within smaller living spaces with fewer facilitating factors, weakening the relationship between HACE facilitator factors and LSA in this study. Additionally, supportive aids for daily living and communication devices included in HACE facilitator factors may have had a minimal impact on LSA, as HR already supports them well.

Regarding physical function, our findings differ from those in independent community-dwelling older adults [25]. While prior studies [25] reported a direct association between motor function and LSA, our results underscore the mediating roles of ADL and IADL. This suggests that interventions prioritizing ADL and IADL, rather than focusing solely on physical function, may be more effective in improving LSA in HR users. Engagement in hobbies has also been reported to influence LSA indirectly via motor function [25]. Hobbies are included in the IADL index FAI [33], which mediated the relationship between motor function and LSA in this study. These findings underscore the importance of promoting IADL alongside HR interventions targeting physical function to enhance LSA.

The structural differences identified between HR users and independent community-dwelling older adults suggest that distinct models are needed to improve LSA in these populations. Unlike independent community-dwelling older adults, HR users may benefit more from support focused on ADL, IADL, and environmental factors.

Additionally, this study found that sex influenced LSA via IADL competence. As sex has been linked to LSA in previous research [8,10], our findings support these reports. The indirect influence of sex on LSA may be explained by the relationship between sex and IADL frequency in Japan, where domestic tasks are performed more frequently by women [45]. The univariate analysis in this study also showed significantly higher FAI scores in women. Since IADL was sex-differentiated among HR users, it can be inferred that sex indirectly influenced LSA via IADL ability.

Another key finding was the indirect influence of SEGE on LSA. As described in Bandura's theory [37], self-efficacy reflects confidence in one's ability to perform activities, shaping perceptions, behaviors, and motivation for outdoor activities. The indirect effect of SEGE on LSA suggests that HR users need confidence in their ability to engage in activities to maintain LSM. Self-efficacy may promote outdoor activities and outings that expand LSM in daily life. The effects of SEGE on FIM-M and FAI further suggest that improving self-efficacy, alongside enhancing ADL and IADL abilities, is essential for increasing LSA. Previous studies [46] have shown that real movement tasks improve LSA and self-efficacy, highlighting the need for HR programs incorporating self-efficacy support.

Collectively, the results of this study suggest that HR programs may improve LSA in HR users by enhancing physical function, ADL and IADL abilities, and self-efficacy through environmental modifications and practice of actual ADL and IADL tasks.

Limitations

This study has four main limitations. First, although it was multicenter, the sample size (n = 105) was relatively small, and participants were drawn from a limited geographical area. Future studies should increase the sample size and include more diverse geographical regions to enhance generalizability.

Second, the parameters used were selected based on previous studies, but cognitive function measures, such as the mini-mental state examination, were not included despite their reported association with LSA. Future studies should incorporate these measures to strengthen the model.

Third, motor function parameters reported to be associated with LSA were not collected. Most previous studies used walking speed, TUG, and one-leg standing tests in highly active community-dwelling older adults [11-14,25,26]. Although important, these measures are challenging to implement in limited home environments and may not be feasible for home-care older adults requiring ADL assistance. The absence of these parameters may partly explain why LSA and physical function were not directly affected in this study. Future research should consider participants’ activity levels and include these motor function parameters, such as knee extension strength and straight leg raising repetition count [47].

Finally, as this is a cross-sectional study, causality cannot be established. Longitudinal studies are needed to clarify influencing factors. The results of this study were obtained in a limited area and cannot be generalized as a system for expanding the LSA of HR users. Although this study has comprehensively assessed HR users based on previous studies and examined factors contributing to the expansion of the LSA, ideally, the study area should be expanded, and HR users should be followed longitudinally to reexamine the findings. Addressing these limitations will allow for a more comprehensive analysis and the development of a more detailed model of LSA in HR users.

## Conclusions

This study found that ADL, IADL, and environmental factors directly influence LSA in HR users, while motor function and psychological factors exert indirect effects. These findings highlight the need for comprehensive HR addressing movement capacity, activity capacity, and environmental factors. Future research should examine a broader population, additional parameters, and longitudinal data to clarify how best to support individuals in HR.
